# Changes in motor cortex excitability associated with temporal repetitive transcranial magnetic stimulation in tinnitus: hints for cross-modal plasticity?

**DOI:** 10.1186/1471-2202-15-71

**Published:** 2014-06-04

**Authors:** Martin Schecklmann, Michael Landgrebe, Tobias Kleinjung, Elmar Frank, Philipp G Sand, Rainer Rupprecht, Peter Eichhammer, Göran Hajak, Berthold Langguth

**Affiliations:** 1 Department of Psychiatry and Psychotherapy, University Regensburg, Universitätsstraße 84, 93053 Regensburg, Germany; 2 Department of Psychiatry, Psychosomatic Medicine and Psychotherapy, Social Foundation Bamberg, Bamberg, Germany; 3 Department of Otorhinolaryngology, University of Zurich, Zurich, Switzerland; 4 Department of Psychiatry, Psychosomatics and Psychotherapy, kbo-Lech-Mangfall-Klinik, Agatharied, Germany

**Keywords:** Transcranial magnetic stimulation, Motor cortex excitability, Motor cortex plasticity, Tinnitus, Cross-modal plasticity, Cortical silent period, Motor threshold, Intracortical inhibition, Intracortical facilitation

## Abstract

**Background:**

Motor cortex excitability was found to be changed after repetitive transcranial magnetic stimulation (rTMS) of the temporal cortex highlighting the occurrence of cross-modal plasticity in non-invasive brain stimulation. Here, we investigated the effects of temporal low-frequency rTMS on motor cortex plasticity in a large sample of tinnitus patients. In 116 patients with chronic tinnitus different parameters of cortical excitability were assessed before and after ten rTMS treatment sessions. Patients received one of three different protocols all including 1 Hz rTMS over the left temporal cortex. Treatment response was defined as improvement by at least five points in the tinnitus questionnaire (TQ). Variables of interest were resting motor threshold (RMT), short-interval intra-cortical inhibition (SICI), intracortical facilitation (ICF), and cortical silent period (CSP).

**Results:**

After rTMS treatment RMT was decreased by about 1% of stimulator output near-significantly in the whole group of patients. SICI was associated with significant changes with respect to treatment response. The group of treatment responders showed a decrease of SICI over the course of treatment, the group of non-responders the reverse pattern.

**Conclusions:**

Minor RMT changes during rTMS treatment do not necessarily suggest the need for systematic re-examination of the RMT for safety and efficacy issues. Treatment response to rTMS was shown to be related to changes in SICI that might reflect modulation of GABAergic mechanisms directly or indirectly related to rTMS treatment effects.

## Background

Tinnitus is associated with neural changes in both the auditory pathway and in non-auditory brain areas [1]. Tinnitus related changes of activity and connectivity in frontal, temporal, parietal, and limbic areas e.g. [2,3] seem to reflect pathologically altered brain networks [4,5]. Based on these findings repetitive transcranial magnetic stimulation (rTMS) was introduced as a treatment approach in tinnitus [6]. However, it turned out that the treatment effects are moderate and associated with high inter-individual variability which raises the need for indicators for effective therapy. Clinical trials of rTMS in tinnitus use typically stimulation of one or both temporal/auditory or temporo-parietal cortices. Recently these protocols were extended by additional stimulation of non-auditory areas such as the frontal cortex to target tinnitus-specific networks more effectively [7,8].

Repetitive transcranial magnetic stimulation (rTMS) as a therapeutic intervention consists of non-invasive repeated stimulation of neocortical areas for hundreds of times per daily sessions via principles of electromagnetic induction. The TMS coil is placed on the subjects’ head over the target area and short-lasting high-intense currents in the coil produce a strong magnetic field (up to 1.5 T), which passes largely undistorted through the scull and induces neuronal depolarisations in the underlying cortical area. rTMS leads to long-term depression- or potentiation-like neuroplastic changes [9]. Low-frequency 1Hz rTMS of motor cortex induces long-term depression as indicated by motor cortex excitability parameters [10]. Effects take place in the directly stimulated cortical area and also in functionally connected remote areas, presumably via cortico-cortical connectivity [11]. For example, stimulation of the dorso-lateral prefrontal cortex is associated with blood flow changes in the anterior cingulate cortex [11,12] or dopamine release in the caudate nucleus [13].

In addition TMS can also be used as a diagnostic tool for the assessment of motor cortex excitability by quantifying contractions of peripheral muscles induced by stimulation of the corresponding motor cortex representation. Assessment of motor cortex excitability in a longitudinal study design before and after a specific therapeutic intervention (e.g. multiple sessions of rTMS) enables to describe treatment-related neuroplastic changes. It has been shown, that rTMS over the temporal cortex can induce changes in motor cortex excitability. In a sham-controlled study 27 healthy subjects showed decreased motor evoked potential amplitudes and delayed cortical silent period (CSP) after five days of 1Hz rTMS of the right temporal cortex [14]. Furthermore, these changes were accompanied by a decrease in glucose metabolism in the stimulated temporal cortex and an increase in cingulate and frontal areas but also in motor cortex. Five days of active low-frequency rTMS over auditory cortex lead to prolongation of the CSP in 18 healthy subjects [15], whereas sham rTMS had not such effects. Clinical benefit of the same protocol in ten patients with tinnitus was positively correlated with changes in short-interval intra-cortical inhibition (SICI), intracortical facilitation (ICF), and CSP [16].

In conclusion, these studies highlight the existence of cross-modal plasticity in rTMS studies in tinnitus or with temporal stimulation. Evidence for functional connectivity between temporal and motor cortex comes from neurophysiologic and brain metabolism studies [17,18]. In tinnitus, the interaction between the sensorimotoric and the auditory system is well established and clinically reflected by somatosensoric modulation of the tinnitus percept [19]. Here, we aimed to investigate the effects of temporal rTMS on different motor cortex excitability parameters in the biggest investigated tinnitus sample so far by means of a retrospective analysis of data obtained in the context of different clinical trials. As only one study in tinnitus with a small sample size was done so far we especially focused on the association of clinical response with change in excitability in the quest for a non-auditory neurophysiological indicator for effective therapy.

## Methods

### Subjects

All participants gave written informed consent after a comprehensive explanation of the procedures. All studies whose data contributed to this analysis were approved by the Ethics Committee at the University of Regensburg. All experiments were conducted in accordance with the last revision of the Declaration of Helsinki.

Measurements of cortical excitability were performed in 116 patients (84 (72.4%) males; 49.2 ± 12.5 (21–83) years) with chronic tinnitus (duration 90 ± 94 (2–476) months). 28 (25.2%) out of 111 patients (data of 5 patients not available) reported a purely left-sided, 21 (18.9%) a purely right-sided tinnitus, and 62 (55.9%) patients described their tinnitus as bilateral or originating within the head. Tinnitus distress was assessed by the German version [20] of the Tinnitus questionnaire (TQ) [21]; TQ baseline scores ranged from 3 to 79 (41 ± 18). Patients suffering from Meniere’s disease, presenting conductive hearing loss or displaying hints of objective tinnitus were not included. 62 patients underwent a complete otologic and audiologic examination including pure tone audiometry, tympanometry, stapedius reflex tests, and otoscopy. Mean hearing level of the audiogram (bilateral hearing thresholds at 0.125, 0.25, 0.5, 1, 2, 3, 4, 6, 8 kHz) was 17 ± 13 (0–61 dB HL). Only patients were included that were eligible for rTMS treatment. Thus, patients with cardiac pacemakers, history of seizures, suspected diagnosis of organic brain damage or any other severe somatic, neurologic, or psychiatric diagnosis were not included.

### Procedures

The therapeutic intervention consisted of 10 rTMS sessions on consecutive weekdays. Treatment effects were evaluated by changes in TQ between the first (day 1) and the last day of treatment (day 12). Motor cortex excitability was examined on the first day before treatment and on the last day after rTMS. We analyzed longitudinal data of 116 patients participating in three different treatments studies [7,22]. Patients received one out of three different active stimulation protocols (2000 stimuli over auditory cortex with 1Hz: n = 68; 4000 stimuli over auditory cortex with 1Hz: n = 26; 2000 stimuli over left frontal cortex with 20Hz followed by 2000 stimuli over auditory cortex with 1Hz: n = 22). Stimulation was set to 110% of the individual resting motor threshold. Localization of the stimulated areas was either done with a neuronavigational system or by using a standard procedure based on the 10–20 system [23]. Recent analyses did not reveal clinically relevant differences in treatment efficacy depending on the used method for coil positioning [7]. In detail, each patient was treated with one protocol lasting ten days and one kind of localization that means that there were sub-groups with different treatments. Data were recorded from 2004 to 2009. All measurements were performed by the same staff which was experienced in the used methods.

For measurement of cortical excitability, participants were seated in a reclining chair. TMS was delivered by two Magstim 200 stimulators (Magstim Co., UK) connected via a Bistim module to a figure-of-eight coil (double-circular-70-mm coil). The coil was held tangential to the skull and with the handle pointing backwards and about 45° away from the midline. The optimal coil position for stimulation was defined as the position above the left motor cortex for eliciting MEP of maximal amplitude in abductor digiti minimi muscle with a slightly supra-threshold stimulus. Once this position was found, it was marked on a scientific head cap and the coil was held in this position by the investigator.

Motor evoked potentials (MEPs) of the abductor digiti minimi of the right hand were recorded with surface electrodes. The analogue signal was registered, band-pass filtered between 20 Hz and 10 kHz, then digitised at a frequency of 5 kHz and analysed off-line. Resting motor threshold (RMT) was determined as the lowest stimulation intensity that evoked in at least four out of eight consecutive trials a MEP of at least 50 μV in the resting abductor digiti minimi [24]. Active motor threshold was defined as the lowest stimulation intensity that evoked in at least four out of eight consecutive trials a MEP of at least 250 μV during isometric contraction of the abductor digiti minimi at about 20% of maximum voluntary contraction. A constant level of voluntary contraction was maintained by audiovisual feedback of the electromyographic (EMG) activity. MEP amplitudes were measured peak-to-peak.

Short-interval intra-cortical inhibition (SICI) and intra-cortical facilitation (ICF) were measured with a paired-pulse TMS protocol [25]. The intensity of the first (conditioning) stimulus was set at 90% of the active motor threshold. The second stimulus was delivered at an intensity that produced MEPs of about 1 mV in the resting abductor digiti minimi. Interstimulus intervals (ISIs) were 2 ms and 15 ms to measure short-interval intra-cortical inhibition (reduction of amplitude) and intracortical facilitation (increase of amplitude), respectively [25]. The conditioned stimuli and the control condition (test pulse alone) were each tested 10 times in a random order (inter-sweep-interval: 4 s). The effect of conditioning stimuli on MEP amplitude at each ISI was determined as the ratio of the average amplitude of conditioned MEP (cMEP) to the average amplitude of unconditioned test MEP (uMEP).

Cortical silent period was measured in 10 trials (stimulus intensity: 150% resting motor threshold; inter-sweep-interval: 5 s) in the moderately active abductor digiti minimi muscle on the non-rectified recording of every individual sweep and then averaged [26]. Participants were instructed to contract this muscle at 30% maximum strength. The onset of the cortical silent period was defined as the end of the MEP when activity dropped consistently below pre-stimulus EMG level. The end of the cortical silent period was defined as first reappearance of voluntary EMG activity. In conclusion, TMS variables of interest were resting motor threshold (RMT), cMEP/uMEP ratio of 2 ms and 15 ms interstimulus intervals (SICI and ICF, respectively), and cortical silent period (CSP).

### Statistics

Statistics are based on retrospective analysis. We were interested if changes of motor cortex excitability were related to rTMS treatment per se and to rTMS induced clinical response. Thus, we did an analysis of variance with treatment as within-subjects factor (first vs. last day of treatment) and treatment response as between-subjects factor (responder vs. non-responder). Treatment response was defined as change in TQ of at least 5 points [27,28]. If an ANOVA revealed significant results, we performed post-hoc t-tests for comparison of responders and non-responders before and after rTMS and for the changes in both groups over time. We were interested in effects of rTMS protocols as potential confounder and repeated these ANOVAs with rTMS protocol as covariate (2000 stimuli temporal vs. 4000 stimuli temporal vs. 2000 stimuli frontal and 2000 temporal) for the significant effects.

As we had four variables of interest (RTM, SICI, ICF, and CSP), significance threshold was set to a Bonferroni corrected level (0.05/4 = 0.0125). For post-hoc tests significance threshold was set to 5%. Statistical analyses were performed with SPSS 18.0.0 (SPSS, USA). As the focus of this analysis were rTMS induced effects on cortical excitability we abstain from reporting treatment efficacy data which have already been published elsewhere [7,22,29,30].

## Results

Firstly, rTMS resulted in a near significant decrease in RMT (reduction by 1% stimulator output) in the whole study population (Table 1). Other parameters of motor cortex excitability remained unchanged. We did not find significant interaction effects of “rTMS protocol” with change in RMT, SICI, ICF, and CSP.

**Table 1:** Descriptive and statistical data of changes in motor cortex excitability in dependence from rTMS intervention and treatment response

		**pre rTMS**	**post rTMS**	**Main effect treatment**	**Main effect response**	**Interaction effect**
resting motor threshold	non-responder	42.6 ± 8.2	41.5 ± 8.5	F = 52.679;	F = 0.062;	F = 0.268;
				df = 1,114;	df = 1,114;	df = 1,114;
	responder	42.8 ± 7.8	42.0 ± 8.0	p = 0.024^+^	p = 0.805	p = 0.606
short-interval intra-cortical inhibition	non-responder	0.58 ± 0.51	0.47 ± 0.28	F = 0.319;	F = 2.323;	F = 6.447;
				df = 1,114;	df = 1,114;	df = 1,114;
	responder	0.41 ± 0.29	0.47 ± 0.33	p = 0.574	p = 0.130	p = 0.012*
intra-cortical facilitation	non-responder	1.68 ± 1.24	1.43 ± 0.74	F = 3.079;	F = 3.474;	F = 2.823;
				df = 1,114;	df = 1,114;	df = 1,114;
	responder	1.32 ± 0.39	1.32 ± 0.36	p = 0.082	p = 0.065	p = 0.096
cortical silent period	non-responder	0.108 ± 0.040	0.107 ± 0.044	F = 0.080;	F = 1.818;	F = 0.179;
				df = 1,114;	df = 1,114;	df = 1,114;
	responder	0.118 ± 0.065	0.123 ± 0.104	p = 0.778	p = 0.180	p = 0.673

*significant at a Bonferroni corrected threshold with p-values displayed uncorrected; ^+^near significant at a Bonferroni corrected threshold with p-values displayed uncorrected.

**Figure 1:**
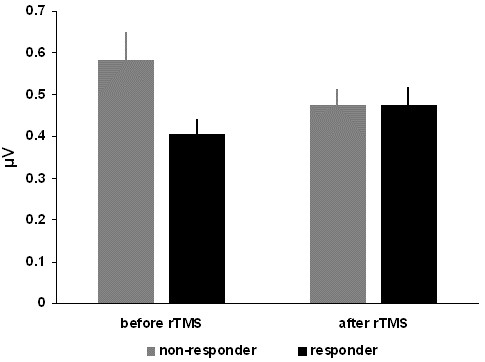
Raw data (mean ± se) of the short-interval intra-cortical inhibition (SICI) for the significant interaction effect of the analysis of variance with the factors rTMS treatment and treatment response.

We found no significant effects of treatment (pre vs. post rTMS). For RMT there was a tendency towards a reduction (of about 1% stimulator output; p = 0.024) but this effect did not reach the Bonferroni-corrected significance level. There was no main effect of group (responder vs. non-responder) either. With respect to the group x treatment interaction there was a significant interaction effect between the change in SICI and the clinical response to rTMS (p = 0.012; Table 1, Figure 1). As post-hoc t-tests indicate, responders showed a reduction of SICI (t = 2.327; df = 56; p = 0.024) and non-responders an increase in SICI (t = 1.737; df = 58; p = 0.088) due to the rTMS intervention. Please note that reduction in SICI is mirrored by a numerical increase of the amplitude ratio between the conditioned stimulus and the test stimulus. Responders significantly differed in their SICI from non-responders before treatment (t = 2.282; df = 114; p = 0.024) whereas there was no difference at the end of treatment (t = 0.002; df = 114; p = 0.999). For the other investigated parameter (RMT, ICF, CSP) the interaction group x treatment was not significant. ANOVAs with rTMS protocol as covariate affirmed the significant findings for RMT (p = 0.091) and SICI (p = 0.013) indicating no influence of the rTMS protocol. Detailed statistic and descriptive data are given in Table 1.

## Discussion

Treatment of 10 days of rTMS over auditory cortex in 116 patients with chronic tinnitus resulted in a near significant, but very small reduction of RMT (1% stimulator output) for the whole group. Since stimulation intensity in most rTMS treatment studies is adjusted to the resting motor threshold (RMT) several earlier studies investigated whether rTMS treatment changes the RMT [5,14-16,31-42]. Findings in these studies are not entirely consistent and it also remains unclear whether observed fluctuations of RMT during the course of rTMS treatment rather reflect variations of cortical excitability or rather an inherent inaccuracy of the measurement procedure of the RMT [43]. Despite these uncertainties there are recommendations to re-measure the RMT over the course of treatment and to adjust the stimulation intensity accordingly [5]. However, the mean reduction by about 1% of the stimulator output in our sample rather suggests that the induced changes in RMT are negligible. Changes in cortical excitability would be meaningful if RMT were shown to be reliable and if stimulation intensity was falling above safe intensity or below effective targeting the brain due to changes of RMT.

Treatment responders as identified by a 5-point reduction in the TQ showed increased SICI at the beginning of the treatment and a reduction over the course of the treatment. Non-responders showed the reverse pattern. Because of the lack of a placebo group we cannot definitively conclude that the observed reduction of SICI during treatment in the responder-group is related to successful rTMS treatment. Theoretically the SICI change could also reflect tinnitus reduction alone, independently of the kind of intervention. However the fact that the responder and non-responder group also differed in their baseline values of SICI makes the latter explanation extremely unlikely.

A relationship between treatment response and a reduction of SICI has not yet been reported before. The discrepancy to previous results may be related to statistical power since the here presented data are from the biggest sample so far. Two studies in healthy controls found no effects of temporal rTMS on SICI [14,15]; in a small sample of tinnitus patients low frequency temporal rTMS increased SICI [16] and also high frequency prefrontal rTMS in patients with major depression increased SICI [31]. However, authors stimulated the left dorso-lateral prefrontal cortex with 20Hz. We used three different treatments all including temporal 1Hz stimulation of the auditory cortex with one treatment including a 20Hz stimulation of left dorso-lateral prefrontal cortex before the temporal stimulation. We did not find any treatment specific effects. One could speculate that opposite effects on SICI after low frequency (decrease of SICI in the present study) and high-frequency rTMS [increase of SICI in 31] may reflect the well-established frequency-dependency of the direction of rTMS treatment effects [10]. However, it is unclear, whether the findings from motor cortex can be directly transferred to non-motor areas since the exact mechanism of the observed cross-modal interaction still remains to be elucidated.

Nevertheless, our findings of altered SICI add further evidence to the notion of cross-modal plasticity of motor cortex in context of stimulation of non-motor areas. Considered mechanisms as indicated by studies in synaesthesia and sensory deprivation (e.g. deafness or blindness) may take place via multi-sensory association areas, via direct cortico-cortical connections, or via subcortical interplay of the corresponding sensory systems at the thalamic level [44-47]. In tinnitus and other neuropsychiatric disorders, the model of thalamocortical dysrhythmia considers altered thalamic activity due to deprived afferent input as the core of the pathology. This altered thalamic activity is generating altered cortical activity which in turn induces symptoms such as phantom perceptions [48]. Based on this concept one could assume that the observed effects on motor cortex excitability in treatment responders are mediated via rTMS induced modulation of thalamic activity. The exact mechanism which underlies the observed cross-modal plasticity effect in this study might be best evaluated by future connectivity studies. Dynamic causal modelling would enable the modelling of the direction of information flow of assumed network hubs. Such studies could elucidate, whether our findings may reflect region-specific changes mediated via specific cross-modal pathways or rather global changes as an unspecific response to local stimulation.

Beside anatomical considerations of cross-modal plasticity, our findings can also be discussed in the context of trans-synaptic chemical signalling. Both antidepressant effects [31] and tinnitus reduction [49], are mediated by the inhibitory-acting γ-aminobutyric acid (GABA). GABA is also involved in motor cortex excitability and plasticity for an overview [50,51]. Especially SICI is mediated by GABAergic interneurons within the primary motor cortex [50,52]. For tinnitus, evidence for GABAergic involvement comes from pharmacological treatments [for an overview 49], animal models [53], and genetic analyses e.g., [54]. Recent studies in animal models of tinnitus identified deficient inhibitory function in input-deprived auditory regions [53]. If low frequency rTMS reduces tinnitus by renormalizing inhibitory function in the auditory cortex, the observed reduction of SICI in treatment responders could reflect the subsequent reduction of a compensatory global inhibitory effort. Following this reasoning the increased baseline SICI may then reflect reduced inhibitory tone in the auditory system. It seems reasonable that rTMS can only exert an effect on tinnitus, if the inhibitory function in the auditory system is altered. In this study we found no effects for other excitability measures. Since RMT, ICF and CSP are rather modulated by other neurotransmitters and -receptors, our findings highlight the role of GABAA mediated neurotansmission in rTMS treatment effects in tinnitus [41,42]. We are well aware that this interpretation is highly speculative and needs to be confirmed by further studies, before further conclusions can be drawn.

On a functional level auditory-motor connectivity might be related to functions of speech or music perception. The motor theory of speech perception links vocal tract gestures to the perception of speech [55]. Beat induction and rhythm perception helps to identify regular patterns in music with the aim to get synchronised by clapping, dancing, or singing [56]. Electrophysiological markers of this transfer may be grounded in the existence of rhythmic oscillatory activity of mu or alpha-like frequencies [57]. Based on these considerations the anatomical, neurotransmitter, and functional association of treatment response to temporal stimulation in tinnitus with SICI highlights the role of auditory-motor connectivity in rTMS treatment of tinnitus. Future clinical approaches might be the identification of responders of daily rTMS based on measurements of SICI changes after single rTMS sessions.

## Conclusions

The counterpoint to the big sample size are the missing control groups and the retrospective analysis. Therefore further prospective studies including control groups are needed before firm conclusions about the specifity of the observed effects for rTMS treatment of tinnitus can be drawn. Moreover consecutive, daily measurements would enable to better characterise the time course of changes in excitability.

Nevertheless the presented data add to the literature that rTMS of non-motor areas can induce changes in motor cortex excitability. RMT changes during rTMS treatment seem to be negligible and do not affirm the potential need for re-evaluation of the stimulation intensity during treatment for efficacy and safety issues. SICI changes might reflect modulation of GABAergic mechanisms directly or indirectly related to rTMS treatment effects.

## Competing interests

No author has to declare competing interest except the receipt of third-party funds from the Tinnitus Research Initiative for the conduction of clinical trials of rTMS in tinnitus.

## Authors’ contributions

MS, ML, TK, EF, PGS, PE, GH, BL have made substantial contributions to conception and design, or acquisition of data, or analysis and interpretation of data. All authors have been involved in drafting the manuscript or revising it critically for important intellectual content. MS, TK, RR, and BL have given final approval of the version to be published. All authors agree to be accountable for all aspects of the work in ensuring that questions related to the accuracy or integrity of any part of the work are appropriately investigated and resolved. All authors read and approved the final manuscript.
